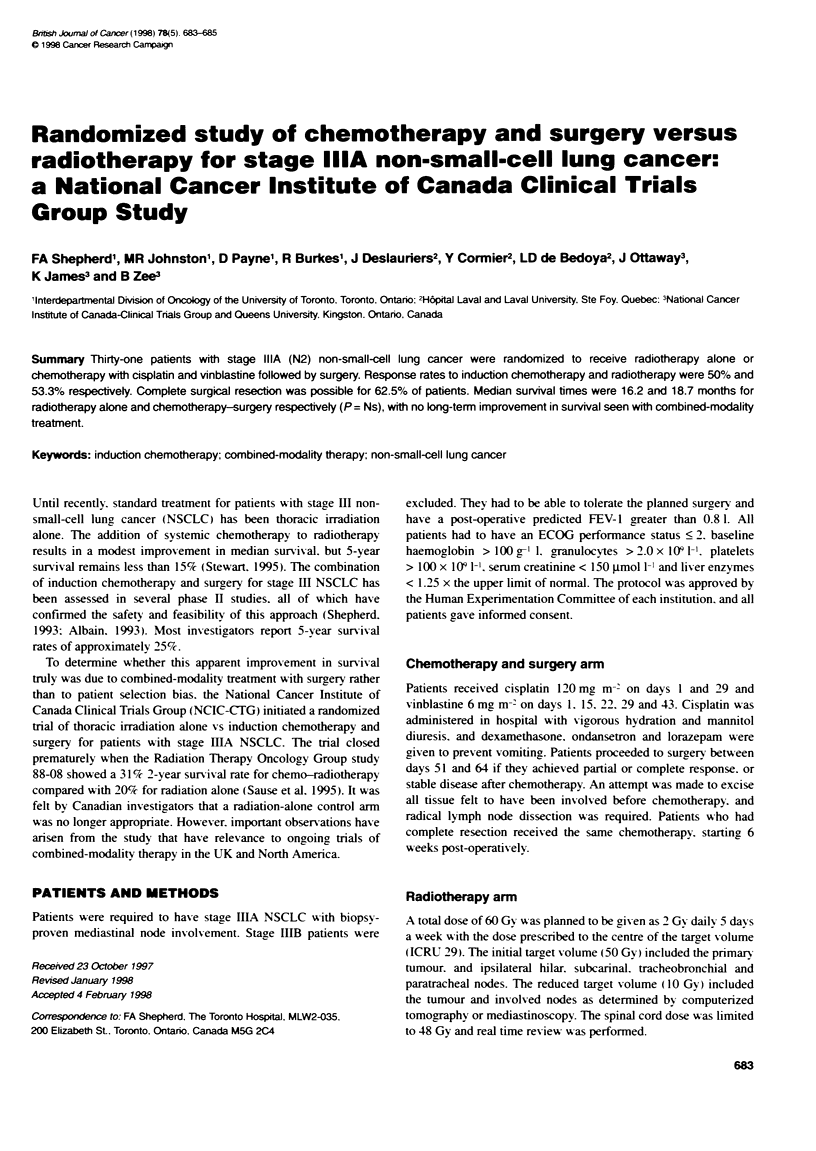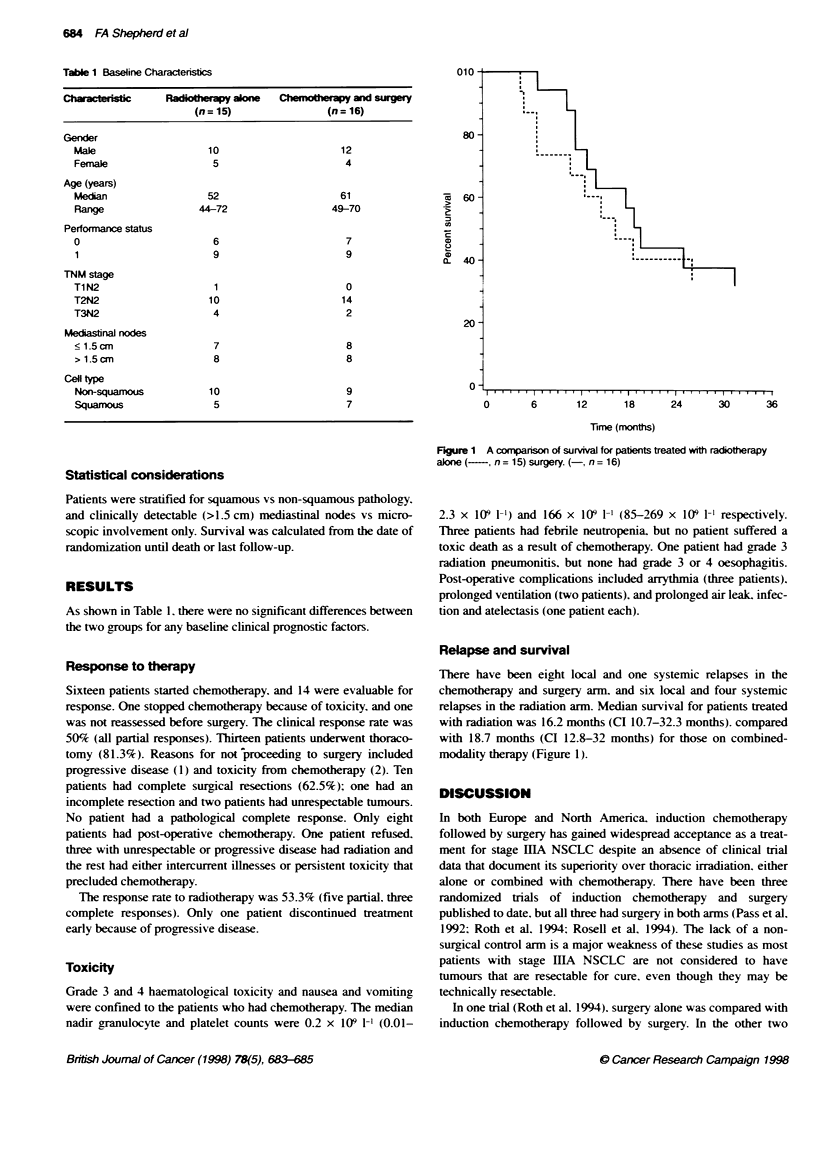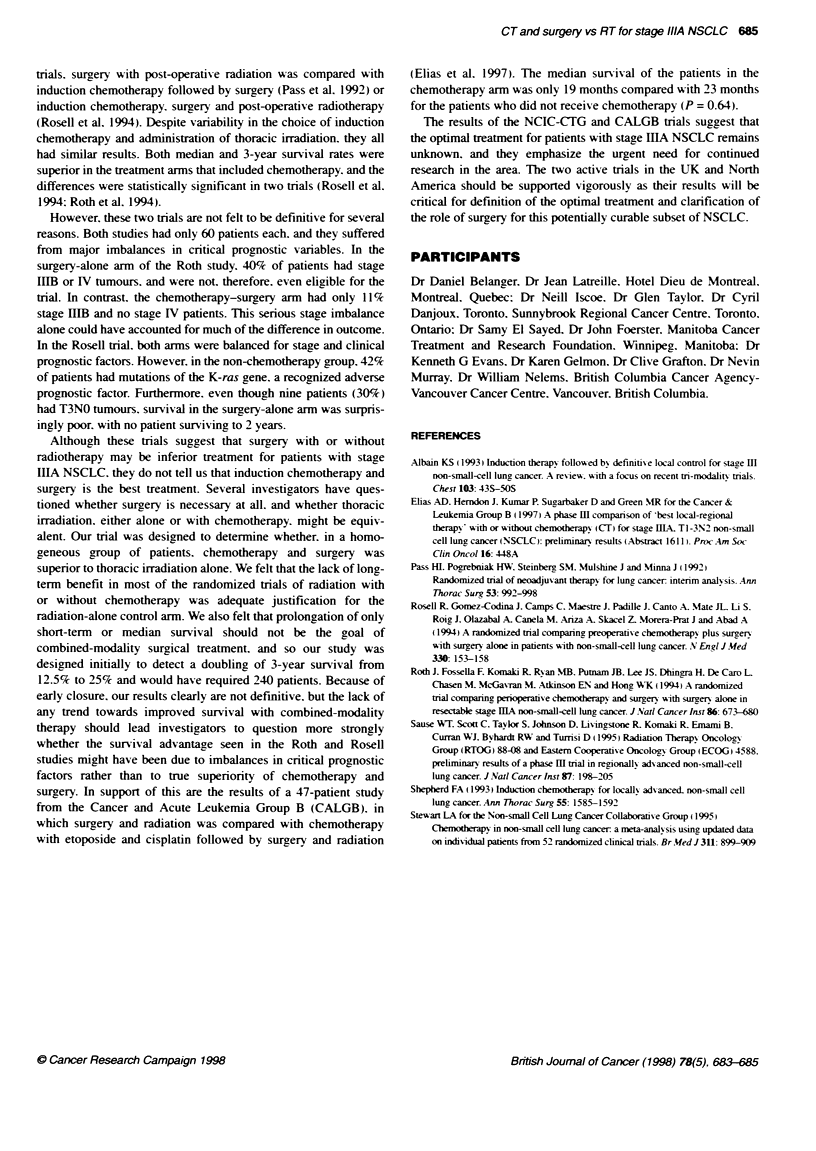# Randomized study of chemotherapy and surgery versus radiotherapy for stage IIIA non-small-cell lung cancer: a National Cancer Institute of Canada Clinical Trials Group Study.

**DOI:** 10.1038/bjc.1998.560

**Published:** 1998-09

**Authors:** F. A. Shepherd, M. R. Johnston, D. Payne, R. Burkes, J. Deslauriers, Y. Cormier, L. D. de Bedoya, J. Ottaway, K. James, B. Zee

**Affiliations:** Interdepartmental Division of Oncology of the University of Toronto, Ontario, Canada.

## Abstract

Thirty-one patients with stage IIIA (N2) non-small-cell lung cancer were randomized to receive radiotherapy alone or chemotherapy with cisplatin and vinblastine followed by surgery. Response rates to induction chemotherapy and radiotherapy were 50% and 53.3% respectively. Complete surgical resection was possible for 62.5% of patients. Median survival times were 16.2 and 18.7 months for radiotherapy alone and chemotherapy-surgery respectively (P = Ns), with no long-term improvement in survival seen with combined-modality treatment.


British Journal of Cancer (1998) 78(5), 683–685
© 1998 Cancer Research Campaign

### Randomized study of chemotherapy and surgery versus radiotherapy for stage IIIA non-small-cell lung cancer: a National Cancer Institute of Canada Clinical Trials Group Study

**FA Shepherd[1], MR Johnston[1], D Payne[1], R Burkes[1], J Deslauriers[2], Y Cormier[2], LD de Bedoya[2], J Ottaway[3], K James[3] and B Zee[3]**

[1]Interdepartmental Division of Oncology of the University of Toronto, Toronto, Ontario; [2]Hôpital Laval and Laval University, Ste Foy, Quebec; [3]National Cancer Institute of Canada-Clinical Trials Group and Queens University, Kingston, Ontario, Canada

**Summary** Thirty-one patients with stage IIIA (N2) non-small-cell lung cancer were randomized to receive radiotherapy alone or chemotherapy with cisplatin and vinblastine followed by surgery. Response rates to induction chemotherapy and radiotherapy were 50% and 53.3% respectively. Complete surgical resection was possible for 62.5% of patients. Median survival times were 16.2 and 18.7 months for radiotherapy alone and chemotherapy–surgery respectively ($P$ = Ns), with no long-term improvement in survival seen with combined-modality treatment.

**Keywords:** induction chemotherapy; combined-modality therapy; non-small-cell lung cancer

Until recently, standard treatment for patients with stage III non-small-cell lung cancer (NSCLC) has been thoracic irradiation alone. The addition of systemic chemotherapy to radiotherapy results in a modest improvement in median survival, but 5-year survival remains less than 15% (Stewart, 1995). The combination of induction chemotherapy and surgery for stage III NSCLC has been assessed in several phase II studies, all of which have confirmed the safety and feasibility of this approach (Shepherd, 1993; Albain, 1993). Most investigators report 5-year survival rates of approximately 25%.

To determine whether this apparent improvement in survival truly was due to combined-modality treatment with surgery rather than to patient selection bias, the National Cancer Institute of Canada Clinical Trials Group (NCIC-CTG) initiated a randomized trial of thoracic irradiation alone vs induction chemotherapy and surgery for patients with stage IIIA NSCLC. The trial closed prematurely when the Radiation Therapy Oncology Group study 88-08 showed a 31% 2-year survival rate for chemo–radiotherapy compared with 20% for radiation alone (Sause et al, 1995). It was felt by Canadian investigators that a radiation-alone control arm was no longer appropriate. However, important observations have arisen from the study that have relevance to ongoing trials of combined-modality therapy in the UK and North America.

### PATIENTS AND METHODS

Patients were required to have stage IIIA NSCLC with biopsy-proven mediastinal node involvement. Stage IIIB patients were excluded. They had to be able to tolerate the planned surgery and have a post-operative predicted FEV-1 greater than 0.8 l. All patients had to have an ECOG performance status ≤ 2, baseline haemoglobin > 100 g$^{-1}$ l, granulocytes > $2.0 \times 10^9$ l$^{-1}$, platelets > $100 \times 10^9$ l$^{-1}$, serum creatinine < 150 μmol l$^{-1}$ and liver enzymes < 1.25 × the upper limit of normal. The protocol was approved by the Human Experimentation Committee of each institution, and all patients gave informed consent.

*Received 23 October 1997*
*Revised January 1998*
*Accepted 4 February 1998*

*Correspondence to:* FA Shepherd, The Toronto Hospital, MLW2-035, 200 Elizabeth St., Toronto, Ontario, Canada M5G 2C4

### Chemotherapy and surgery arm

Patients received cisplatin 120 mg m$^{-2}$ on days 1 and 29 and vinblastine 6 mg m$^{-2}$ on days 1, 15, 22, 29 and 43. Cisplatin was administered in hospital with vigorous hydration and mannitol diuresis, and dexamethasone, ondansetron and lorazepam were given to prevent vomiting. Patients proceeded to surgery between days 51 and 64 if they achieved partial or complete response, or stable disease after chemotherapy. An attempt was made to excise all tissue felt to have been involved before chemotherapy, and radical lymph node dissection was required. Patients who had complete resection received the same chemotherapy, starting 6 weeks post-operatively.

### Radiotherapy arm

A total dose of 60 Gy was planned to be given as 2 Gy daily 5 days a week with the dose prescribed to the centre of the target volume (ICRU 29). The initial target volume (50 Gy) included the primary tumour, and ipsilateral hilar, subcarinal, tracheobronchial and paratracheal nodes. The reduced target volume (10 Gy) included the tumour and involved nodes as determined by computerized tomography or mediastinoscopy. The spinal cord dose was limited to 48 Gy and real time review was performed.

Table 1 Baseline Characteristics

| Characteristic | Radiotherapy alone (n = 15) | Chemotherapy and surgery (n = 16) |
|---|---|---|
| Gender | | |
| Male | 10 | 12 |
| Female | 5 | 4 |
| Age (years) | | |
| Median | 52 | 61 |
| Range | 44–72 | 49–70 |
| Performance status | | |
| 0 | 6 | 7 |
| 1 | 9 | 9 |
| TNM stage | | |
| T1N2 | 1 | 0 |
| T2N2 | 10 | 14 |
| T3N2 | 4 | 2 |
| Mediastinal nodes | | |
| ≤ 1.5 cm | 7 | 8 |
| > 1.5 cm | 8 | 8 |
| Cell type | | |
| Non-squamous | 10 | 9 |
| Squamous | 5 | 7 |

Figure 1 A comparison of survival for patients treated with radiotherapy alone (------, n = 15) surgery. (—, n = 16)

### Statistical considerations

Patients were stratified for squamous vs non-squamous pathology, and clinically detectable (>1.5 cm) mediastinal nodes vs microscopic involvement only. Survival was calculated from the date of randomization until death or last follow-up.

### RESULTS

As shown in Table 1, there were no significant differences between the two groups for any baseline clinical prognostic factors.

### Response to therapy

Sixteen patients started chemotherapy, and 14 were evaluable for response. One stopped chemotherapy because of toxicity, and one was not reassessed before surgery. The clinical response rate was 50% (all partial responses). Thirteen patients underwent thoracotomy (81.3%). Reasons for not proceeding to surgery included progressive disease (1) and toxicity from chemotherapy (2). Ten patients had complete surgical resections (62.5%); one had an incomplete resection and two patients had unrespectable tumours. No patient had a pathological complete response. Only eight patients had post-operative chemotherapy. One patient refused, three with unrespectable or progressive disease had radiation and the rest had either intercurrent illnesses or persistent toxicity that precluded chemotherapy.

The response rate to radiotherapy was 53.3% (five partial, three complete responses). Only one patient discontinued treatment early because of progressive disease.

### Toxicity

Grade 3 and 4 haematological toxicity and nausea and vomiting were confined to the patients who had chemotherapy. The median nadir granulocyte and platelet counts were $0.2 \times 10^9$ $l^{-1}$ (0.01–$2.3 \times 10^9$ $l^{-1}$) and $166 \times 10^9$ $l^{-1}$ (85–$269 \times 10^9$ $l^{-1}$ respectively. Three patients had febrile neutropenia, but no patient suffered a toxic death as a result of chemotherapy. One patient had grade 3 radiation pneumonitis, but none had grade 3 or 4 oesophagitis. Post-operative complications included arrythmia (three patients), prolonged ventilation (two patients), and prolonged air leak, infection and atelectasis (one patient each).

### Relapse and survival

There have been eight local and one systemic relapses in the chemotherapy and surgery arm, and six local and four systemic relapses in the radiation arm. Median survival for patients treated with radiation was 16.2 months (CI 10.7–32.3 months), compared with 18.7 months (CI 12.8–32 months) for those on combined-modality therapy (Figure 1).

### DISCUSSION

In both Europe and North America, induction chemotherapy followed by surgery has gained widespread acceptance as a treatment for stage IIIA NSCLC despite an absence of clinical trial data that document its superiority over thoracic irradiation, either alone or combined with chemotherapy. There have been three randomized trials of induction chemotherapy and surgery published to date, but all three had surgery in both arms (Pass et al, 1992; Roth et al, 1994; Rosell et al, 1994). The lack of a non-surgical control arm is a major weakness of these studies as most patients with stage IIIA NSCLC are not considered to have tumours that are resectable for cure, even though they may be technically resectable.

In one trial (Roth et al, 1994), surgery alone was compared with induction chemotherapy followed by surgery. In the other two

© Cancer Research Campaign 1998

trials, surgery with post-operative radiation was compared with induction chemotherapy followed by surgery (Pass et al, 1992) or induction chemotherapy, surgery and post-operative radiotherapy (Rosell et al, 1994). Despite variability in the choice of induction chemotherapy and administration of thoracic irradiation, they all had similar results. Both median and 3-year survival rates were superior in the treatment arms that included chemotherapy, and the differences were statistically significant in two trials (Rosell et al, 1994; Roth et al, 1994).

However, these two trials are not felt to be definitive for several reasons. Both studies had only 60 patients each, and they suffered from major imbalances in critical prognostic variables. In the surgery-alone arm of the Roth study, 40% of patients had stage IIIB or IV tumours, and were not, therefore, even eligible for the trial. In contrast, the chemotherapy–surgery arm had only 11% stage IIIB and no stage IV patients. This serious stage imbalance alone could have accounted for much of the difference in outcome. In the Rosell trial, both arms were balanced for stage and clinical prognostic factors. However, in the non-chemotherapy group, 42% of patients had mutations of the K-*ras* gene, a recognized adverse prognostic factor. Furthermore, even though nine patients (30%) had T3N0 tumours, survival in the surgery-alone arm was surprisingly poor, with no patient surviving to 2 years.

Although these trials suggest that surgery with or without radiotherapy may be inferior treatment for patients with stage IIIA NSCLC, they do not tell us that induction chemotherapy and surgery is the best treatment. Several investigators have questioned whether surgery is necessary at all, and whether thoracic irradiation, either alone or with chemotherapy, might be equivalent. Our trial was designed to determine whether, in a homogeneous group of patients, chemotherapy and surgery was superior to thoracic irradiation alone. We felt that the lack of long-term benefit in most of the randomized trials of radiation with or without chemotherapy was adequate justification for the radiation-alone control arm. We also felt that prolongation of only short-term or median survival should not be the goal of combined-modality surgical treatment, and so our study was designed initially to detect a doubling of 3-year survival from 12.5% to 25% and would have required 240 patients. Because of early closure, our results clearly are not definitive, but the lack of any trend towards improved survival with combined-modality therapy should lead investigators to question more strongly whether the survival advantage seen in the Roth and Rosell studies might have been due to imbalances in critical prognostic factors rather than to true superiority of chemotherapy and surgery. In support of this are the results of a 47-patient study from the Cancer and Acute Leukemia Group B (CALGB), in which surgery and radiation was compared with chemotherapy with etoposide and cisplatin followed by surgery and radiation (Elias et al, 1997). The median survival of the patients in the chemotherapy arm was only 19 months compared with 23 months for the patients who did not receive chemotherapy ($P = 0.64$).

The results of the NCIC-CTG and CALGB trials suggest that the optimal treatment for patients with stage IIIA NSCLC remains unknown, and they emphasize the urgent need for continued research in the area. The two active trials in the UK and North America should be supported vigorously as their results will be critical for definition of the optimal treatment and clarification of the role of surgery for this potentially curable subset of NSCLC.

### PARTICIPANTS

Dr Daniel Belanger, Dr Jean Latreille, Hotel Dieu de Montreal, Montreal, Quebec; Dr Neill Iscoe, Dr Glen Taylor, Dr Cyril Danjoux, Toronto, Sunnybrook Regional Cancer Centre, Toronto, Ontario; Dr Samy El Sayed, Dr John Foerster, Manitoba Cancer Treatment and Research Foundation, Winnipeg, Manitoba; Dr Kenneth G Evans, Dr Karen Gelmon, Dr Clive Grafton, Dr Nevin Murray, Dr William Nelems, British Columbia Cancer Agency-Vancouver Cancer Centre, Vancouver, British Columbia.

### REFERENCES

Albain KS (1993) Induction therapy followed by definitive local control for stage III non-small-cell lung cancer. A review, with a focus on recent tri-modality trials. *Chest* **103**: 43S–50S

Elias AD, Herndon J, Kumar P, Sugarbaker D and Green MR for the Cancer & Leukemia Group B (1997) A phase III comparison of 'best local-regional therapy' with or without chemotherapy (CT) for stage IIIA, T1-3N2 non-small cell lung cancer (NSCLC): preliminary results (Abstract 1611). *Proc Am Soc Clin Oncol* **16**: 448A

Pass HI, Pogrebniak HW, Steinberg SM, Mulshine J and Minna J (1992) Randomized trial of neoadjuvant therapy for lung cancer: interim analysis. *Ann Thorac Surg* **53**: 992–998

Rosell R, Gomez-Codina J, Camps C, Maestre J, Padille J, Canto A, Mate JL, Li S, Roig J, Olazabal A, Canela M, Ariza A, Skacel Z, Morera-Prat J and Abad A (1994) A randomized trial comparing preoperative chemotherapy plus surgery with surgery alone in patients with non-small-cell lung cancer. *N Engl J Med* **330**: 153–158

Roth J, Fossella F, Komaki R, Ryan MB, Putnam JB, Lee JS, Dhingra H, De Caro L, Chasen M, McGavran M, Atkinson EN and Hong WK (1994) A randomized trial comparing perioperative chemotherapy and surgery with surgery alone in resectable stage IIIA non-small-cell lung cancer. *J Natl Cancer Inst* **86**: 673–680

Sause WT, Scott C, Taylor S, Johnson D, Livingstone R, Komaki R, Emami B, Curran WJ, Byhardt RW and Turrisi D (1995) Radiation Therapy Oncology Group (RTOG) 88-08 and Eastern Cooperative Oncology Group (ECOG) 4588, preliminary results of a phase III trial in regionally advanced non-small-cell lung cancer. *J Natl Cancer Inst* **87**: 198–205

Shepherd FA (1993) Induction chemotherapy for locally advanced, non-small cell lung cancer. *Ann Thorac Surg* **55**: 1585–1592

Stewart LA for the Non-small Cell Lung Cancer Collaborative Group (1995) Chemotherapy in non-small cell lung cancer: a meta-analysis using updated data on individual patients from 52 randomized clinical trials. *Br Med J* **311**: 899–909

© Cancer Research Campaign 1998